# Plasma Proteomic Biomarkers Relating to Alzheimer’s Disease: A Meta-Analysis Based on Our Own Studies

**DOI:** 10.3389/fnagi.2021.712545

**Published:** 2021-07-21

**Authors:** Liu Shi, Noel J. Buckley, Isabelle Bos, Sebastiaan Engelborghs, Kristel Sleegers, Giovanni B. Frisoni, Anders Wallin, Alberto Lléo, Julius Popp, Pablo Martinez-Lage, Cristina Legido-Quigley, Frederik Barkhof, Henrik Zetterberg, Pieter Jelle Visser, Lars Bertram, Simon Lovestone, Alejo J. Nevado-Holgado

**Affiliations:** ^1^Department of Psychiatry, University of Oxford, Oxford, United Kingdom; ^2^Department of Psychiatry and Neuropsychology, School for Mental Health and Neuroscience, Alzheimer Centrum Limburg, Maastricht University, Maastricht, Netherlands; ^3^Alzheimer Center, VU University Medical Center, Amsterdam, Netherlands; ^4^Reference Center for Biological Markers of Dementia (BIODEM), Institute Born-Bunge, Department of Biomedical Sciences, University of Antwerp, Antwerp, Belgium; ^5^Department of Neurology, Universitair Ziekenhuis Brussel and Center for Neurociences (C4N), Vrije Universiteit Brussel, Brussels, Belgium; ^6^Complex Genetics Group, VIB Center for Molecular Neurology, VIB, Antwerp, Belgium; ^7^Institute Born-Bunge, Department of Biomedical Sciences, University of Antwerp, Antwerp, Belgium; ^8^Department of Psychiatry, University of Geneva, Geneva, Switzerland; ^9^Institute of Neuroscience and Physiology, Department of Psychiatry and Neurochemistry, The Sahlgrenska Academy at the University of Gothenburg, Mölndal, Sweden; ^10^Hospital de la Santa Creu i Sant Pau, Barcelona, Spain; ^11^Department of Psychiatry, University Hospital of Lausanne, Lausanne, Switzerland; ^12^Geriatric Psychiatry, Department of Mental Health and Psychiatry, Geneva University Hospitals, Geneva, Switzerland; ^13^CITA-Alzheimer Foundation, San Sebastian, Spain; ^14^Kings College London, London, United Kingdom; ^15^The Systems Medicine Group, Steno Diabetes Center, Gentofte, Denmark; ^16^Department of Radiology and Nuclear Medicine, VU University Medical Center, Amsterdam, Netherlands; ^17^UCL Institutes of Neurology and Healthcare Engineering, London, United Kingdom; ^18^Clinical Neurochemistry Laboratory, Sahlgrenska University Hospital, Mölndal, Sweden; ^19^UK Dementia Research Institute at UCL, London, United Kingdom; ^20^Department of Neurodegenerative Disease, UCL Institute of Neurology, London, United Kingdom; ^21^Lübeck Interdisciplinary Platform for Genome Analytics, University of Lübeck, Lübeck, Germany; ^22^Department of Psychology, University of Oslo, Oslo, Norway; ^23^Janssen R&D, High Wycombe, United Kingdom

**Keywords:** Alzheimer’s disease (AD), diagnosis, blood biomarkers, meta-analysis, proteomic

## Abstract

**Background and Objective**: Plasma biomarkers for the diagnosis and stratification of Alzheimer’s disease (AD) are intensively sought. However, no plasma markers are well established so far for AD diagnosis. Our group has identified and validated various blood-based proteomic biomarkers relating to AD pathology in multiple cohorts. The study aims to conduct a meta-analysis based on our own studies to systematically assess the diagnostic performance of our previously identified blood biomarkers.

**Methods**: To do this, we included seven studies that our group has conducted during the last decade. These studies used either Luminex xMAP or ELISA to measure proteomic biomarkers. As proteins measured in these studies differed, we selected protein based on the criteria that it must be measured in at least four studies. We then examined biomarker performance using random-effect meta-analyses based on the mean difference between biomarker concentrations in AD and controls (CTL), AD and mild cognitive impairment (MCI), MCI, and CTL as well as MCI converted to dementia (MCIc) and non-converted (MCInc) individuals.

**Results**: An overall of 2,879 subjects were retrieved for meta-analysis including 1,053 CTL, 895 MCI, 882 AD, and 49 frontotemporal dementia (FTD) patients. Six proteins were measured in at least four studies and were chosen for meta-analyses for AD diagnosis. Of them, three proteins had significant difference between AD and controls, among which alpha-2-macroglobulin (A2M) and ficolin-2 (FCN2) increased in AD while fibrinogen gamma chain (FGG) decreased in AD compared to CTL. Furthermore, FGG significantly increased in FTD compared to AD. None of the proteins passed the significance between AD and MCI, or MCI and CTL, or MCIc and MCInc, although complement component 4 (CC4) tended to increase in MCIc individuals compared to MCInc.

**Conclusions**: The results suggest that A2M, FCN2, and FGG are promising biomarkers to discriminate AD patients from controls, which are worthy of further validation.

## Introduction

Currently, the diagnosis of Alzheimer’s disease (AD) clinically is based on clinical examination, patient and carer interview, and structural or glucose metabolism imaging (McKhann et al., 2011). The limitation of this approach is that a significant proportion of AD patients have their diagnosis changed after a measure of amyloid either by positron emission tomography (PET) or lumbar puncture (Barthel and Sabri, 2017), or post-mortem studies (Beach et al., 2012; Selvackadunco et al., 2019). Furthermore, syndrome-based AD diagnosis is too late given that AD pathology happens 10 or even 20 years before a clinical symptom appears (Jack et al., 2010). Measuring biomarkers through PET or in cerebrospinal fluid (CSF) can close this gap not only for clinical research but also to define AD pathophysiologically (Jack et al., 2018). However, the expense, invasiveness, and dependence on relevant infrastructure limit their utility in clinical practice (de Almeida et al., 2011; Lista et al., 2013).

Blood-based biomarkers represent a less invasive and potentially cost-effective approach for the diagnosis and classification of AD processes. Numerous studies have sought plasma biomarkers relevant to AD and great progress has been made during the past several decades (Blennow, 2017). For example, recent studies demonstrated that AD hallmarks in plasma such as Aβ42/40, p-tau 181, and 217 can predict brain pathology with high accuracy, further adding evidence that they can be used as a non-invasive approach for the diagnosis and prognosis of AD (Nakamura et al., 2018; Karikari et al., 2020, 2021; Mattsson-Carlgren et al., 2020; Thijssen et al., 2020; Janelidze et al., 2021). Despite these advances, great variability has been observed in blood biomarker validity in individual studies. A recent meta-analysis (Koychev et al., 2021) showed that analytical assays have played an important role in deciding the reliability of detection of AD hallmarks in blood and further research is needed to further validate their use as screening tools.

Apart from hallmarks in blood, an increasing number of studies (Baird et al., 2015; Zetterberg and Burnham, 2019; Manzine et al., 2020), including those by ourselves (Thambisetty et al., 2010b; Kiddle et al., 2012; Westwood et al., 2016), have found that a range of proteins in plasma might act as biomarkers. In our review published in 2018 (Shi et al., 2018), we summarized some of the main findings and approaches taken in the studies that we have conducted during the last decade. Since then, we have further validated these identified biomarkers in two large independent cohorts including over 1,500 individuals. Taking all these studies together, this study aims to conduct a meta-analysis to systematically examine the level of individual biomarkers in blood as diagnostic tools to discriminate AD patients from healthy subjects.

## Materials and Methods

### Study Selection

Studies were selected for meta-analysis based on two inclusion criteria: (1) the study must include a group of AD patients and CTL (control) or MCIc (MCI converted to AD) and MCInc to (MCI non-converted to AD) conduct pare-wise meta-analysis; and (2) Using a quantitative method to assess biomarker concentrations in blood (such as ELISA and Luminex xMAP). As a result, seven studies were included in this study including European Medical Information Framework (EMIF1000; Westwood et al., 2020), AddNeuroMed (Hye et al., 2014), EMIF500 (Westwood et al., 2018), VU University Medical Center (VUMC; Westwood et al., 2018), Australian Imaging, Biomarkers and Lifestyle Flagship Study of Ageing (AIBL; Ashton et al., 2015), University of California, San Francisco, Memory and Aging Center (UCSF; Ashton et al., 2015) and GE (Westwood et al., 2018) study. Among these studies, the diagnosis of AD-type dementia was based on the National Institute of Neurological and Communicative Disorders and Stroke–Alzheimer’s Disease and Related Disorders Association criteria (McKhann et al., 1984). Of note, the EMIF1000 and EMIF500 datasets included different subjects. As proteins measured in these studies differed, a protein was selected for meta-analysis based on the criteria that it must be measured in at least four studies.

### Meta-Analysis

All analyses were completed using the R package meta for (Viechtbauer, 2010). An effect size was defined by the mean difference of biomarker concentration in the two groups such as AD vs. the control group. Values below 0 indicate that the mean concentration of biomarker was higher in the disease group, otherwise indicating lower in the disease group. The variance of difference was estimated using the delta method. Given that these studies are not exactly identical in the characteristics of the included samples, we, therefore, chose random-effect models to calculate each biomarker separately. Random-effect models assume that the true effect size varies across studies based on a normal distribution with mean μ and variance τ2 (heterogeneity), indicating each study has its true effect size θi. Estimated effect sizes yi is the study-specific sampling variance that is caused by measurement error, assuming to be normally distributed with mean θi and variance vi. Confidence intervals were used to assess the significance of the estimated overall effect size. The alpha level was set to 5% and confidence and prediction intervals were 95% for all tests reported below unless it is specified.

## Results

### Description of Studies

We included seven studies that our group has conducted during the last decade for meta-analysis. These studies measured candidate biomarkers for AD pathology-related processes using either Luminex xMAP or ELISA. The number of sample size and proteins measured in each study are shown in Table 1. Overall, the meta-analysis contained 2,879 individuals including 1,053 healthy individuals, 895 mild cognitive impairment (MCI) patients, 882 AD patients, and 49 frontotemporal dementia (FTD). Furthermore, MCI individuals included 216 subjects who subsequently converted to dementia within 3 years and 539 non-converted. Details of proteins were shown in Supplementary Table 1.

**Table 1:** Description of subjects and the included studies in this meta-analysis.

Study	Sample size						Number of proteins
	CTL (*n* = 1,053)	MCI (*n* = 895)	MCInc (*n* = 539)	MCIc (*n* = 216)	AD (*n* = 882)	FTD (*n* = 49)
EMIF1000 (Westwood et al., 2020)	408	400	237	103	192	/	25
AddNeuroMed (Hye et al., 2014)	452	220	169	51	476	/	30
EMIF500 (Westwood et al., 2018)	97	235	/	/	162	/	21
VUMC (Westwood et al., 2018)	43	17	/	/	22	/	9
AIBL (Ashton et al., 2015)	49	23	12	10	6	/	20
UCSF (Ashton et al., 2015)	4	/	/	/	24	49	8
GE (Westwood et al., 2018)	/	/	121	52	/	/	34

*AD, Alzheimer’s disease; CTL, control; MCI, mild cognitive impairment; MCIc, MCI converted to AD; MCInc, MCI non-converted to AD; FTD, frontotemporal dementia; EMIF, European Medical Information Framework; VUMC, VU University Medical Center; AIBL, Australian Imaging, Biomarkers and Lifestyle Flagship Study of Ageing; UCSF, University of California, San Francisco, Memory and Aging Center*.

### Meta-Analyses of Blood Biomarkers for AD Diagnosis

**Figure 1:**
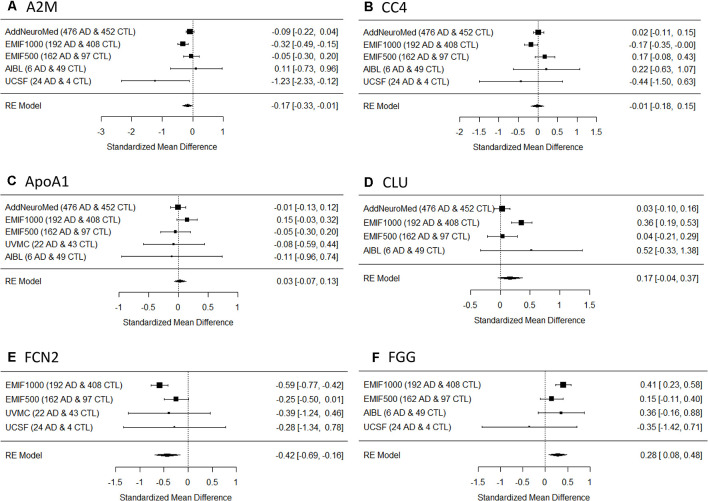
Forest plot of six proteins between Alzheimer’s disease (AD) and controls. RE, random effect; **(A)** A2M, alpha-2-macroglobulin; **(B)** CC4, complement component 4; **(C)** ApoA1, apolipoprotein A-I; **(D)** CLU, clusterin; **(E)** FCN2, ficolin-2; **(F)** FGG, fibrinogen gamma chain; EMIF, European Medical Information Framework; VUMC, VU University Medical Center; AIBL, Australian Imaging, Biomarkers and Lifestyle Flagship Study of Ageing; UCSF, University of California, San Francisco, Memory and Aging Center.

**Figure 2:**
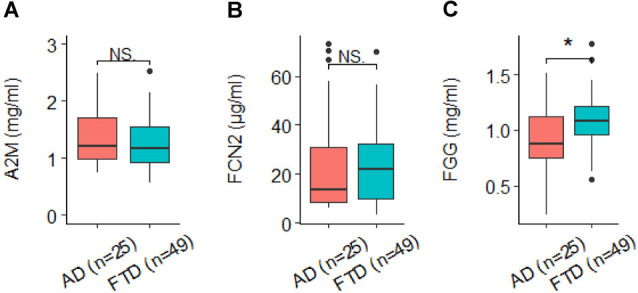
**(A)** A2M, alpha-2-macroglobulin; **(B)** FCN2, ficolin-2; **(C)** FGG, fibrinogen gamma chain. Box plot of three proteins’ expression between AD and frontotemporal dementia (FTD). NS., not significant. *p < 0.05.

Six proteins were measured in at leastfour studies and were chosen for meta-analysis for AD diagnosis. They were alpha-2-macroglobulin (A2M), complement component 4 (CC4), apolipoprotein A-I (ApoA1), clusterin (CLU), ficolin-2 (FCN2), and fibrinogen gamma chain (FGG). Meta-analysis showed that three proteins had significant differences between AD and controls, among which A2M and FCN2 increased in AD (Figures 1A,E) while FGG decreased in AD (Figure 1F). In comparison, the other three proteins did not show significant differences between AD and controls (Figures 1B–D). None of the proteins passed the significance between AD and MCI or MCI and controls. Furthermore, three proteins (A2M, FCN2, and FGG) were also measured in FTD individuals in the UCSF study. Pairwise comparisons showed that no significant difference was found for A2M (Figure 2A) or FCN2 (Figure 2B) between FTD and AD while FGG significantly increased in FTD compared to AD (Figure 2C).

### Meta-Analyses of Blood Biomarkers for Predicting MCI Conversion

**Figure 3:**
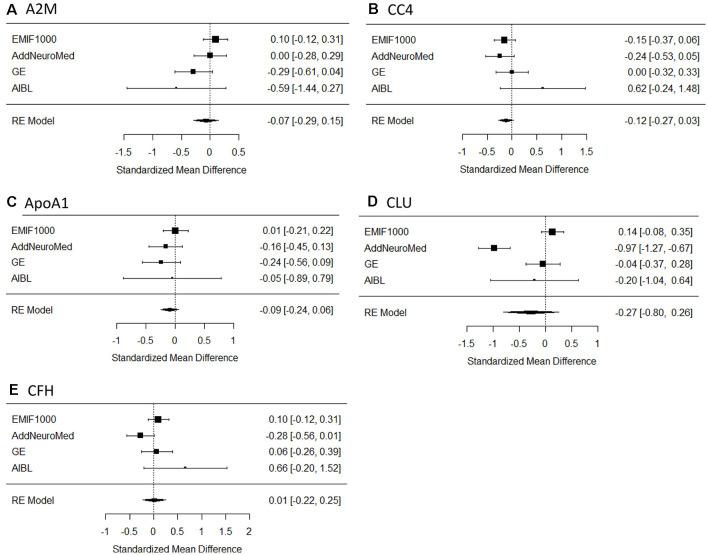
Forest plot of five proteins between MCIc and MCInc. MCI, mild cognitive impairment; MCIc, MCI converted to AD; MCInc, MCI non-converted to AD; RE, random effect; **(A)** A2M, alpha-2-macroglobulin; **(B)** CC4, complement component 4; **(C)** ApoA1, apolipoprotein A-I; **(D)** CLU, clusterin; **(E)** CFH, complement factor H; EMIF, European Medical Information Framework; AIBL, Australian Imaging, Biomarkers and Lifestyle Flagship Study of Ageing.

Overall, five proteins were measured in at least four studies in MCIc and MCInc and were chosen for meta-analysis. The proteins were A2M, ApoA1, CC4, CLU, and complement factor H (CFH). Results showed that none of the proteins reached the significance between MCIc and MCInc (Figures 3A,C–E), although CC4 tended to increase in MCIc individuals compared to MCInc (Figure 3B).

## Discussion

In this meta-analysis study, we aimed to evaluate the diagnostic value of our previously identified blood-based biomarkers for AD. We found that A2M and FCN2 increased in AD while FGG decreased in AD compared to CTL. Furthermore, FGG significantly increased in FTD compared to AD, indicating it might be specific for AD diagnosis, while further validation in large independent cohorts is needed. In contrast, none of the proteins passed the significance between AD and MCI or MCI and controls from the meta-analysis. This might be caused by the fact that MCI included both MCIc subjects and MCInc subjects. As MCIc and MCInc are different groups, combining them as a single group might lead to insignificant results.

Our initial discovery-phase studies demonstrated that plasma A2M, FCN2, and FGG were closely associated with AD pathology (Shi et al., 2018). For example, all the three markers were associated with amyloid deposition (Kiddle et al., 2012; Ashton et al., 2015; Westwood et al., 2016, 2017). Furthermore, FCN2 and FGG were related to brain atrophy and rate of cognitive decline (Thambisetty et al., 2010a, 2011; Sattlecker et al., 2014). Apart from our own studies, other studies found that these proteins were biologically relevant to the disease process. For example, it was found that A2M was localized to diffuse amyloid plaques in AD brains (Kovacs, 2000). From the genetic evidence, A2M gene DNA polymorphisms caused increased accumulation of amyloid plaques in the brain of AD patients (Kovacs, 2000). Ficolins are activators of the lectin complement pathway (Fujita et al., 2004). Ficolin-3 (FCN3) is another member of the ficolin family, sharing approximately 50% amino acid sequence homology with FCN2 (Kilpatrick and Chalmers, 2012). It has been found that FCN3 is related to insulin resistance and diabetes (Li et al., 2008; Chen et al., 2012; Zhang et al., 2016). This is very interesting because there is a close relationship between diabetes and AD (Janson et al., 2004; Talbot et al., 2012). Fibrinogen was found to accumulate along with AD pathology progresses (Ryu and McLarnon, 2009) and co-deposits with amyloid plaques in brain tissue (Klohs et al., 2012). Furthermore, it has been found that fibrinogen binds to amyloid, enhancing amyloid aggregation and fibrillization (Ahn et al., 2010). All the evidence further confirms the relevance of A2M, FCN2, and FGG in AD pathogenesis, indicating they are promising biomarkers for AD diagnosis.

Current findings on plasma biomarkers have generated new enthusiasm in the blood biomarker field, particularly plasma neurofilament light (NfL), Aβ42/40, p-tau 181 and 217, and glial fibrillary acidic protein (GFAP; Nakamura et al., 2018; Karikari et al., 2020, 2021; Mattsson-Carlgren et al., 2020; Sugarman et al., 2020; Thijssen et al., 2020; Chatterjee et al., 2021; Cicognola et al., 2021; Clark et al., 2021; Janelidze et al., 2021). However, the samples in these studies were clinical trial populations, the performance of these biomarkers in community-based populations was much worse. For example, one study in ADNI reported that among people who are cognitively impaired, plasma p-tau181 distinguished amyloid-positives with a moderate area under curve (AUC) of 0.67 (Tosun et al., 2021), much lower than the AUCs of 0.77–0.91 reported in some memory clinic cohorts (Karikari et al., 2020; Thijssen et al., 2020). Likewise, another study using a small cohort found that plasma p-tau181 discriminated 20 cognitively normal amyloid-positive people from 31 amyloid-negative people with an AUC of only 0.67 (Barthélemy et al., 2020). Therefore, these biomarkers cannot stand alone in predicting AD diagnosis or AD pathology.

Compared to AD core markers, the three markers (A2M, FCN2, and FGG) obtained in this study had relatively lower AUC. For example, our previous study showed that plasma A2M distinguished AD from controls with an AUC of 0.61 (Hye et al., 2006). The AUC of FCN2 to classify amyloid status was 0.64 (Westwood et al., 2020). The combination of FGG with age achieved an AUC of 0.69 in discriminating amyloid status (Ashton et al., 2015). Despite lower AUC compared to AD core markers, the three proteins can add extra value as they reflect different aspects of the disease. Therefore, building algorithms combining AD core markers with additional factors such as demographic information as well as other potential blood-based biomarkers are needed to add diagnostic value. Here, our meta-analysis showed that A2M, FCN2, and FGG are good candidates for AD diagnosis and worthy of further validation.

Our study has two main limitations. First, the diagnosis of AD in our previous studies was based on clinical diagnosis instead of using the ATN framework. Therefore, future studies are needed to confirm these biomarkers in discriminating pathologically confirmed AD-type dementia. Second, this study is not a meta-analysis of the published literature but rather a meta-analysis of the data we generated on our own. However, because our studies used the same analytical platforms to measure these biomarkers, the meta-analysis results were not affected by the difference in platforms. Furthermore, there is a higher probability of effective utility in practice as the current methods (Luminex xMAP and ELISA) can be easily adopted in clinic settings.

In conclusion, we demonstrate that A2M, FCN2, and FGG in blood have the potential use as screening tools to diagnose AD along with other promising blood biomarkers. Further validation in bigger, more epidemiologically sampled populations that better represent the community populations are needed.

## Data Availability Statement

The datasets will be made available by the authors to qualified researchers upon reasonable request. Requests to access the datasets should be directed to the corresponding author.

## Ethics Statement

The studies involving human participants were reviewed and approved by all 23 medical Ethics Committees. The patients/participants provided their written informed consent to participate in this study. Written informed consent was obtained from the individual(s) for the publication of any potentially identifiable images or data included in this article.

## Author Contributions

LS carried out data analysis and interpretation as well as drafted the manuscript. All authors contributed to the article and approved the submitted version.

## Conflict of Interest

SL is named as an inventor on biomarker intellectual property protected by Proteome Sciences and Kings College London unrelated to the current study and within the past 5 years has advised for Optum labs, Merck, SomaLogic and been the recipient of funding from AstraZeneca and other companies *via* the IMI funding scheme. SL is employed by company Janssen. HZ has served at scientific advisory boards for Alector, Denali, Roche Diagnostics, Wave, Samumed, Siemens Healthineers, Pinteon Therapeutics and CogRx, has given lectures in symposia sponsored by Cellectricon, Fujirebio, Alzecure and Biogen, and is a co-founder of Brain Biomarker Solutions in Gothenburg AB (BBS), which is a part of the GU Ventures Incubator Program (all unrelated to this study). AL has served at scientific advisory boards of Fujirebio Europe, Eli Lilly, Novartis, Nutricia and Otsuka and is the inventor of a patent on synaptic markers in CSF (all unrelated to this study). JP has served at scientific advisory boards of Fujirebio Europe, Eli Lilly and Nestlé Institute of Health Sciences, all unrelated to this study. SE has received unrestricted research grants from Janssen Pharmaceutica and ADx Neurosciences and has served at scientific advisory boards of Biogen, Eisai, Novartis, Nutricia/Danone and Roche, all unrelated to this study. The remaining authors declare that the research was conducted in the absence of any commercial or financial relationships that could be construed as a potential conflict of interest.
