# Supplementary material for: Plasma Proteomic Biomarkers Relating to Alzheimer’s Disease: A Meta-Analysis Based on Our Own Studies
Source: Front Aging Neurosci. 2021 Jul 21;13:712545. doi: 10.3389/fnagi.2021.712545 (PMC8335587; doi:10.3389/fnagi.2021.712545)
Supplement: Supplementary file 1 [file Table_1.DOCX]

**Supplementary Table 1. Description of proteins in each study.**

| **Study** | **Proteins** |
| --- | --- |
| EMIF1000 | **25 proteins:** FCN2, FGG, CysC, CLU, B2M, AGP, CP, A2M, ApoA1, ApoC3, ApoE, TTR, CFH, CRP  A1AT, PEDF, SAP, CC4, BDNF, CathepsinD, sICAM1, RANTES, NCAM, sVCAM1 and PAI1 |
| AddNeuroMed | **30 proteins:** ApoA1, ApoC3, ApoE, TTR, CFH, A2M, CRP, A1AT, PEDF, SAP, CC4, BDNF, sVCAM1, sICAM1, CathepsinD, RANTES, PAI1, NCAM, AB40, AB42, sRage, NSE, BDNF, CP, AGP, SAP, Haptoglobin, CysC, CLU and B2M |
| EMIF500 | **21 proteins:** A2M, ApoA, ApoC4, C4b, CFHR1, FCN2, FGG, IgKappa, IGHG3, TRF, A1AT, CC4, CLU, CysC, ApoC3, AGP, sICAM1, PEDF, TTR, RANTES and ApoA1 |
| VUMC | **9 proteins:** APOC4, FCN2, SAA4, FGb, ApoA1, APOA4, C4BPA, IGHG3 and TRF |
| AIBL | **20 proteins:** A2M, APO(a), ApoA1, APOL1, CC3, CC4, CFB, CFH, CFH-R1, Fga, FGG, GPX-3, GSN, HPT, HPX, HRG, TRF, APOAIV, C1s and CLU |
| UCSF | **8 proteins:** CFHR1, HRG, FCN2, APOC4, A2M, C3, CC4 and FGG |
| GE | **34 proteins:** B2M, CLU, CysC, CP, AGP, BDNF, CathepsinD, sICAM1, RANTES, NCAM, sVCAM1, PAI1, A2M, ApoA1, ApoC3, ApoE, TTR, CFH, CRP, A1AT, PEDF, SAP, CC4, AB40, NSE, A2M, ApoA, APOC4, CFHR1, FCN2, FGG, TRF, IgKappa and PPY |

EMIF, European Medical Information Framework; VUMC, VU University Medical Center; AIBL, Australian Imaging, Biomarkers and Lifestyle Flagship Study of Ageing; UCSF, University of California, San Francisco, Memory and Aging Center.
